# Positive sentiments in early academic literature on DeepSeek: a cross-disciplinary mini review

**DOI:** 10.3389/frai.2025.1725853

**Published:** 2026-01-12

**Authors:** Yuxing He, Angie Giangan, Nam Vu, Casey Watters

**Affiliations:** 1 Faculty of Law, Bond University, Robina, QLD, Australia; 2 Independent researcher, Robina, QLD, Australia; 3 Centre for Logistics, Procurement and Supply Chain Management, Cranfield School of Management, Cranfield University, Cranfield, United Kingdom

**Keywords:** artificial intelligence, censorship, Chinese AI, deep learning, DeepSeek, large language models (LLM), natural language processing (NLP), neural networks

## Abstract

DeepSeek is a free and self-hostable large language model (LLM) that recently became the most downloaded app across 156 countries. As early academic literature on ChatGPT was predominantly critical of the model, this mini-review is interested in examining how DeepSeek is being evaluated across academic disciplines. The review analyzes available articles with DeepSeek in the title, abstract, or keywords, using the VADER sentiment analysis library. Due to limitations in comparing sentiment across languages, we excluded Chinese literature in our selection. We found that Computer Science, Engineering, and Medicine are the most prominent fields studying DeepSeek, showing an overall positive sentiment. Notably, Computer Science had the highest mean sentiment and the most positive articles. Other fields of interest included Mathematics, Business, and Environmental Science. While there is substantial academic interest in DeepSeek’s practicality and performance, discussions on its political or ethical implications are limited in academic literature. In contrast to ChatGPT, where all early literature carried a negative sentiment, DeepSeek literature is mainly positive. This study enhances our understanding of DeepSeek’s reception in the scientific community and suggests that further research could explore regional perspectives.

## 1 Introduction

Founded in 2023, DeepSeek is a Chinese-developed AI chatbot that has emerged as a major competitor to ChatGPT. Functionally, DeepSeek mirrors many of ChatGPT’s core capabilities. It offers direct responses to user queries and can retrieve real-time information from the internet. However, it was not until January 2025, following the release of its R1 reasoning model, that the company gained global fame. DeepSeek ranked first in the most downloaded app across over 156 countries, exceeding ChatGPT for the first time (Field, 2025).

Since 2023, DeepSeek has released a series of generative AI models that have continually improved in both capabilities and performance. The progressive launch of its models, which include:

- DeepSeek Coder, an open-source coding model released in November 2023
- DeepSeek LLM, a general-purpose model released in December 2023
- DeepSeek – V2, an efficiency-focused general-purpose model released in May 2024
- DeepSeek –Coder-V2, designed for complex coding tasks and released in July 2024
- DeepSeek-V3 adopted a mixture-of-experts architecture to handle complex tasks and improve accuracy, and was released in December 2024
- DeepSee-R1, an improvement on the V3 model focused on advanced reasoning, was released in January 2025
- Janus – Pro – 7B, a model focusing on understanding and generating images, released in January 2025; and
- DeepSeek -R1-0528, an updated version of R1 integrating agentic AI, was released in May 2025.

DeepSeek focuses on developing open source LLMs, which means, according to a working definition put forth by the Open Source Initiative, that it grants users the freedom to use the AI for any purpose without seeking permission, to study how the system works and inspects its components, to modify the AI including changing the output and to share the AI for others to use (Williams and O’Donnell, 2024; Open Source Initiative O. S. I, 2024; Bansemer and Miller, 2025). This self-hosting capacity offers significant privacy advantages, especially for institutional users, developers, and enterprises seeking greater control over data governance and customization. Although the model cannot be considered fully open source since its training data have not been made entirely available, the data weights are downloadable. They can, therefore, be run locally, allowing users to protect their own data (Gibney and Pachocki, 2025) something important as companies increasingly rely on data in decision making and disclosure (Wan and Watters, 2021; Abdullah et al., 2025).

The emergence of open AI sources, such as DeepSeek, represents a pivotal shift in the AI ecosystem. By making powerful LLMs and coding agents openly accessible, DeepSeek lowers the barriers to entry for individuals, academic institutions, and smaller companies that may not have the resources to train models from scratch. This democratization of advanced AI technologies fosters a more inclusive innovation environment, encouraging global participation in AI development beyond the confines of a few dominant tech firms (Sapkota et al., 2025). This transparency cultivates deeper understanding and accelerates collective learning.

While much of the public discourse has focused on the general user experience or industry use of AI (Yang et al., 2025), this study explicitly examines how DeepSeek is being evaluated within academic disciplines. There is a significant body of literature on using LLMs to determine sentiment (Kiziltepe et al., 2025; Mouthami et al., 2025; Shah et al., 2025; Muhammad and Rospocher, 2025), and a growing body of literature examines public sentiment towards LLM models (Demirel et al., 2025; Islam et al., 2025; Katta, 2025), including DeepSeek (Tubishat et al., 2025; Hossain, 2025; Santosa et al., 2025; Lalupanda et al., 2025). However, few articles examine academic sentiment regarding ChatGPT and early LLM models (Tao and Shen, 2025; Watters and Lemanski, 2023; Twinomurinzi and Gumbo, 2023; Mostafa and Beshir, 2025). This is the first study purely examining academic research on DeepSeek.

While early academic literature on ChatGPT was universally critical across disciplines (Watters and Lemanski, 2023), it remains to be seen how scholars are responding to DeepSeek in its initial phase of academic reception. It is therefore important to investigate not only the overall sentiment expressed in early studies of DeepSeek but also whether differences in evaluation emerge across academic fields. In the next section, this review outlines the methodology, including the inclusion policies for the literature. Then, section three details the findings and a discipline-based discussion of the sentiment towards DeepSeek. Finally, the review concludes in section 4.

## 2 Methodology: literature search strategy and inclusion criteria

To answer the research question, this paper first needed to identify the existing literature within various academic disciplines. The Scopus database was selected because it is a well-recognized indexing database commonly used in reviews (Kusuma et al., 2024; Culbert et al., 2025; Hartanto et al., 2024) and because it already categorizes publications by discipline, thereby reducing the risk of author bias in article categorization.

A search was conducted on May 25, 2025, for all Scopus-indexed publications with ‘DeepSeek’ in the title, abstract, or as a keyword. This search provided 144 documents. Conference papers, editorials and other non-article formats were excluded, leaving 80 articles, of which 69 were in English (see Table 1). Then, the articles available through the Bond University Library were downloaded, and their sentiment was analyzed using VADER, a sentiment analysis tool within NLTK (Natural Language Toolkit), a Python library. VADER is commonly used for sentiment analysis in academic research (Asthana et al., 2024; Kalamkar and Sharma, 2024; Singla et al., 2024; Kumar et al., 2024). Although one of its benefits is analysing short texts, it is also used in standard academic texts and literature (Vinodini, 2023; Youvan, 2024). Approaches to sentiment analysis can be broadly categorized as lexicon-based (Zhuo et al., 2024; Kumar et al., 2024; Catelli et al., 2022), as is VADER, or machine/deep learning based. ML models, BERT being one of the most common, require training on a dataset and are often fine-tuned (Taye et al., 2025; Rhomrasi et al., 2025; Alaparthi and Mishra, 2021; Goud and Garg, 2025; Culbert et al., 2025). This makes them more nuanced but less reproducible and more subjective. Here, we used the integrated lexicon for VADER, thereby providing a more reproducible and objective approach, which is valuable for analyzing topics that may be controversial. VADER also employs a single, unified lexicon, which did not require fine-tuning but was initially validated against sample texts. Despite these benefits, the decreased language nuance in the lexicon approach can result in more extreme results.

**Table 1:** Scopus literature on DeepSeek.

Discipline	Total	Sentiment	Citations
Computer Science	28	Mean 0.866 Positive 0.933	Yang et al. (2025), Alghamdi and Mostafa (2025), Bevara et al. (2025), Li M. et al. (2025), Liu Y. et al. (2025), Bai et al. (2025), Chen Y. Q. et al. (2025), Marcaccini et al. (2025a), Fernandes et al. (2025), Jiao et al. (2025), Spennemann (2025), Roumeliotis et al. (2025), Rasool et al. (2025), Li J. et al. (2025), Du et al. (2025), Fei et al. (2025), Deng Z. et al., 2025, Xiong et al., 2025, Mavridis et al. (2025), Alsaif et al. (2025), Zyda, 2025, Valmeekam et al. (2025), Liu Y. J. et al. (2025), Zhang et al., 2025, Ben Saad et al., 2025, Franzoni et al. (2024), Munley et al. (2024), Cassano et al. (2024)
Engineering	16	Mean 0.7501 Positive 0.87	Yang et al. (2025), Li M. et al. (2025), Dong et al. (2025), Jiang et al. (2025), Bai et al. (2025), Fernandes et al. (2025), Jiao et al. (2025), Rhomrasi et al. (2025), Spennemann (2025), Roumeliotis et al. (2025), Peng et al. (2025), Fei et al. (2025), Deng Z. et al. (2025), Xiong et al. (2025), Okaiyeto et al. (2025), Cassano et al. (2024)
Medicine	15	Mean 0.749 Positive 0.875	Cai et al. (2025), Uldin et al. (2025), Prasad et al. (2025), Zhou J. et al. (2025), Patil et al. (2025), Zhou M. et al. (2025), Mccoy and Perlis (2025), Chen C. C. et al. (2025), Marcaccini et al. (2025b), Bhattacharya et al. (2025), Ali (2025), Zeng et al. (2025), Zhou H. et al. (2025), Deng M. et al. (2025), Alsammarraie and Househ (2025)
Social Sciences	10	Mean 0.9997	Mizumoto and Teng (2025), Sroyprapai et al. (2025), Bevara et al. (2025), Liu Y. et al. (2025), Chen Y. Q. et al. (2025), Pal and Ray (2025), Spennemann (2025), Tewari (2025), Arnal (2025)
Environmental Sci	4	Mean 0.9995	Liu Y. et al. (2025), Dong et al. (2025), Jiang et al. (2025), Peng et al. (2025)
Econ & Finance	4	Mean 0.9999	Yang (2025), Moravec et al. (2025), Pal and Ray (2025), Tewari (2025)
Business	4	Mean 0.9999	Dwivedi (2025), Moravec et al. (2025), Spennemann (2025), Saleh (2025)
Mathematics	3	Mean 1.0	Deng Z. et al. (2025), Xiong et al. (2025), Zhang et al. (2025)
Dentistry	3	Mean 0.997	Kaygisiz and Teke (2025), Yilmaz et al. (2025), Diniz-Freitas and Diz-Dios (2025)
BioChem/Genetics	3	Mean 0.9998	Seth et al. (2025), Sandmann et al. (2025), Tordjman et al. (2025)
Categories with 2 or less articles	12	Mean 0.999	Si et al. (2025), Yang et al. (2025), Shao et al. (2025), Jia et al. (2025), Liu Y. et al. (2025), Peters and Chin-Yee (2025), Chen Y. Q. et al. (2025), Chen C. C. et al. (2025), Fei et al. (2025), Xie et al. (2025), Ben Saad et al. (2025), Okaiyeto et al. (2025)
Word usage heat map 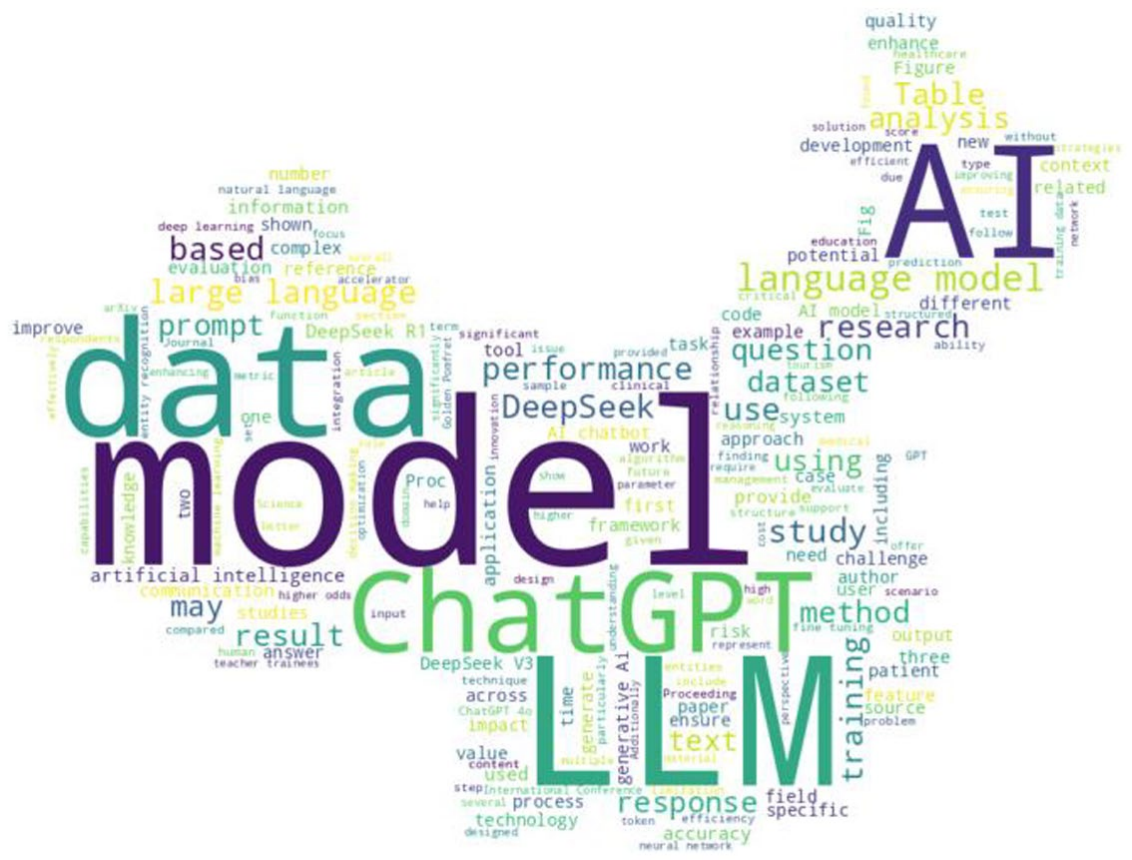			

**Figure 1:**
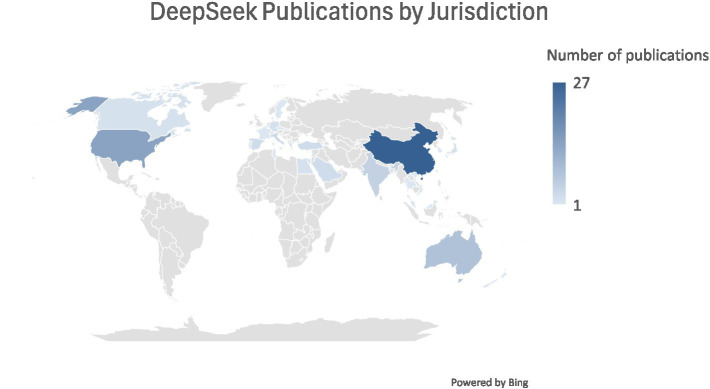
DeepSeek publications by jurisdiction.

Sentiment is represented on a scale from −1 to 1, with −1 signifying entirely negative sentiment, zero being neutral, and 1 signifying entirely favorable treatment. The mean sentiment was calculated for available articles in each academic discipline, with the percentage of positive articles also calculated for disciplines with 15 or more articles. This data is presented in Table 1. Furthermore, the articles were examined for their topics, and a heatmap of the words used in the articles was also included. Lastly, we examined the countries where authors are located to understand the distribution of authorship and its potential impact on sentiment. This was presented in Figure 1. Articles that were written by multiple authors in different countries or that are classified in more than one discipline are included in all applicable categories.

This research aims to provide a structured overview of how scholars are engaging with DeepSeek, a major non-Western AI model. This fills a gap in AI-related literature analysis that is still focused on models developed by Western companies (e.g., Google, OpenAI). In addition, by identifying how different academic fields evaluate DeepSeek, this research would potentially reveal any disciplinary biases, priorities, and blind spots. However, one significant limitation of the research is that it excludes the Chinese language literature. Languages each have their own styles and subtleties that lexicons and models attempt to encapsulate. However, no model or lexicon in one language can be the objective equivalent of one in another. To avoid bias, sentiment can therefore not be reliably compared across languages. To avoid bias, this study focuses on English literature, with a future study of Chinese literature recommended.

## 3 The findings and discussion

### 3.1 Findings

Unlike ChatGPT, which received negative sentiment in the early literature, DeepSeek has been met with a more positive reception. However, this may be less a result of sentiment specific to DeepSeek but rather a reflection of increasing comfort with the role of artificial intelligence, and large language models in particular. Table 1 presents the mean sentiment for articles across various academic disciplines, along with the percentage of positive articles for disciplines with 15 or more articles. A word map is also used to illustrate the most common terms across all disciplines in the articles Figure 1 shows the geographic distribution of authorship. Unsurprisingly, the jurisdictions with the most authored papers were China, followed by the United States.

### 3.2 Implications and research gap

#### 3.2.1 Disciplinary analysis

The analysis produces an overall positive sentiment toward DeepSeek, with no discipline demonstrating a mean sentiment below 0.74, and most clustering close to 1.0 (see Table 1). This reflects a generally favorable perception of DeepSeek in early academic literature. From the analysis of disciplinary differences, three disciplines - Computer Science (28 articles), Engineering (16 articles), and Medicine (15 articles) - account for over half of the data. This suggests that DeepSeek is most impactful in technical and scientific fields. However, these patterns should be interpreted with caution. The total dataset includes only 69 articles, and many fields are represented by a relatively small number of publications. The limited size of the corpus may constrain the generalizability of disciplinary trends. Nevertheless, existing literature generally agrees that by making the model’s intermediate reasoning steps transparent, DeepSeek supports more trustworthy and verifiable outputs when it launched its reasoning model. This capability is especially valuable in computer science and technical research settings, where understanding how a conclusion is reached is often as important as the conclusion itself.

Among these three fields, it is interesting to note that (within the limits of the dataset) Computer Science demonstrated the highest mean sentiment (0.866) as well as the most significant proportion of positive articles (93.3%), reflecting a possible strong appreciation for DeepSeek’s open-source nature, computational efficiency, and potential for technical experimentation and fine-tuning models. This sentiment is also likely influenced by DeepSeek’s contributions to the coding domain. The release of DeepSeek Coder in late 2023 marked a significant milestone as China’s first open-source coding-specific model, offering a competitive alternative to international models such as OpenAI’s Codex and Google’s Gemini. In contrast, when ChatGPT was first introduced, it lacked strong coding capabilities, an area that has since become a key benchmark in LLM performance. DeepSeek’s strong early positioning in this space may have bolstered its reception in Computer Science research communities. Meanwhile, Engineering and Medicine had slightly lower mean sentiment scores (0.7501 and 0.749, respectively), with 87.5% of articles in both fields showing positive evaluations. This relative moderation, unsurprisingly, reflects discipline-specific concerns, as Engineering research tends to approach new tools like DeepSeek primarily as components within larger socio-technical systems, where questions of reliability, robustness, integration with existing infrastructure, and life-cycle maintenance are central (Breznická et al., 2023; Baxter and Sommerville, 2011).

Medicine, on the other hand, operates within an explicitly risk-averse and heavily regulated framework. Clinical disciplines typically require strong empirical evidence, clear regulatory guidance, and well-defined accountability before integrating new technologies into practice (Pham, 2025). In this context, known limitations of large language models (such as hallucinations, lack of guaranteed accuracy, difficulties in ensuring explainability, and concerns over patient data privacy and medico-legal liability) carry considerable weight. Even if DeepSeek’s reasoning model and open-source architecture are viewed as promising for clinical decision support, education, or documentation, medical authors may be more inclined to emphasise potential harms and ethical constraints. Taken together, these disciplinary norms help explain why Engineering and Medicine, while still largely positive, exhibit slightly more cautious sentiment toward DeepSeek than Computer Science, where experimentation, coding support, and model fine-tuning are more immediately aligned with core research practices.

Several disciplines reported near-perfect sentiment averages. Small sample sizes in these disciplines may artificially inflate sentiment scores, since a few highly favorable articles can significantly skew the average in the absence of a critical mass. These findings should therefore be treated as preliminary observations rather than conclusive indicators of disciplinary enthusiasm.

#### 3.2.2 Word cloud analysis

Through the word cloud, the most frequently occurring terms included “model,” “data,” “ChatGPT,” “LLM,” “AI,” “DeepSeek,” and “language” (see Word Cloud in Table 1). This concentration of technical terminology suggests that the prevailing academic interest centers on the architecture, performance, and training methods of DeepSeek, often in comparison to other large language models (LLMs), such as ChatGPT. This is also reflected in the prominence of the term “ChatGPT” recorded in the map. Furthermore, the frequent appearance of terms such as “training,” “dataset,” “accuracy,” and “output” reinforces the focus on model benchmarking and quantitative evaluation.

A significant portion of the high-frequency terms identified in the heatmap (e.g., “use,” “case,” “question,” “tool,” “method,” “task,” “context,” “study”) suggests a practical orientation in the literature. This indicates that DeepSeek is not only examined as a technological development but also evaluated for its utility across various academic contexts, including research assistance, content generation, and information retrieval. The presence of domain-specific terms, such as “healthcare,” “education,” “clinical,” “communication,” and “patient,” indicates DeepSeek’s interdisciplinary reach. Its perceived usefulness spans technical disciplines such as computer science and engineering, as well as applied domains like medicine, education, and business. This supports the earlier findings from sentiment analysis that DeepSeek is being actively explored across diverse academic fields, albeit with different evaluative priorities.

Notably absent from the heat map are terms related to ethics, regulation, censorship, or governance (e.g., “bias,” “trust,” “privacy,” “surveillance”). This suggests that while technical and application-oriented discussions are well-developed, critical engagement with the political or ethical implications of DeepSeek - such as its alignment with state censorship norms or privacy concerns - remains limited in the current literature. As noted in the introduction, DeepSeek has been criticized for censorship. This stands in contrast to the earlier literature on ChatGPT, which often advocated for what might be considered a form of censorship due to concerns about bias. A key difference is that the literature addressing bias risk for ChatGPT focuses primarily on the training data. One of the challenges in training early LLMs is accessing data to train the LLM. The best sources, such as news and academic papers, are often subject to copyright and behind paywalls. The discussion and criticisms of censorship primarily focus on the limitations of post-training answers that large language models can provide. However, the training dataset also has a significant impact, and the decisions over which data to include or exclude can always be criticized. Fortunately, multiple models can now be used simultaneously for users who want different perspectives or seek to mitigate bias. Additionally, following the approach of DeepSeek, many thinking models display the reasoning behind the model’s answers. This empowers the user but requires more active engagement with the language model than many casual users may be willing to undertake.

#### 3.2.3 Geographical analysis

In terms of the geographic distribution of publications, unsurprisingly, China accounts for the highest number of publications (27), reflecting its role as the originator and primary developer of DeepSeek. Beyond simple proximity, this domestic prominence is closely tied to China’s broader strategy of fostering domestic AI capability and digital sovereignty, in which home-grown large language models are positioned as strategic assets that can reduce dependence on US-controlled technologies and infrastructure (Chang et al., 2025). The United States emerges as the second most active jurisdiction, suggesting substantial international interest in evaluating Chinese AI models, driven by comparative research agendas or the open-source availability of DeepSeek’s model architecture. However, this interest is not merely technical. DeepSeek has been framed in Western policy and media discourse as both a symbol of China’s accelerating AI capabilities and a potential inflection point in the global AI race, prompting reassessments of Chinese progress in open-source and reasoning-capable models. At the same time, DeepSeek has been linked to concerns over national security, data privacy, and information control, which leads to proposed and actual restrictions on its use within government and critical sectors, and public warnings about censorship and disinformation risks (Freifeld, 2025). These geopolitical dynamics help to explain why early English-language scholarship is concentrated in China and the US.

India, Australia, Canada, Germany, and the United Kingdom each contribute between two and five publications, while isolated single contributions appear from several other regions. This pattern suggests widespread but uneven global engagement with DeepSeek. Linguistic and academic-network factors provide a more convincing explanation than geopolitics. In medium- and low-volume countries, geopolitical rivalry is also less central to how DeepSeek is perceived. Unlike in the United States, where DeepSeek can be framed as a potential strategic competitor to domestic models, many of these jurisdictions do not position DeepSeek as a direct national rival in their own AI industrial strategies. For them, DeepSeek tends to appear as one tool among many in a wider ecosystem dominated by US and European providers. This weakens the explanatory power of a purely geopolitical lens. It has been found that DeepSeek systematically refuses or reshapes answers on politically sensitive topics related to China, and that sensitive content can appear in internal reasoning while being suppressed or rewritten in the final output (Qiu et al., 2025). For researchers and practitioners in many countries, this makes DeepSeek less attractive as a general-purpose information tool, since its outputs on politically or historically contested questions are perceived as incomplete or biased. Outside China, DeepSeek is therefore more likely to be used for comparative benchmarking, bias and censorship audits, or technical experimentation, rather than as a trusted knowledge source. This helps to explain why, despite its technical appeal and open-weight availability, sustained scholarly engagement remains relatively limited in many parts of the world.

## 4 Conclusion

Early adopters of DeepSeek are typically researchers who are already positively inclined toward generative AI and who primarily frame the system as a pragmatic tool for enhancing efficiency, rather than as an object of ethical or political concern. When DeepSeek is used mainly for summarization, drafting, translation, or data handling, authors tend to focus on whether it “works” in practice and improves workflows, rather than interrogating its broader implications. In addition, critical and normative analyses of emerging technologies usually appear later in the publication cycle than technical reports or methodological case studies, as evidence of harms, biases, or structural effects takes time to accumulate.

A similar lag can be observed with respect to legal and regulatory concerns. Potentially contentious issues - such as responsibility for erroneous outputs, the use of copyrighted or sensitive data in training, data protection and cross-border transfers, or the legal status of AI-assisted authorship - require careful doctrinal and empirical analysis. These questions typically take longer to surface in the literature than methodological case studies or technical evaluations, and they often appear in specialized legal or policy venues that may fall outside the initial corpus. As a result, early work is more likely to present DeepSeek as a useful, low-cost, open-source resource than to interrogate its compliance with data protection regimes, intellectual property law, or emerging AI regulation. Taken together, these dynamics help to explain why early English-language literature on DeepSeek is characterized by very high sentiment scores and limited critical engagement, especially in fields where the model is used instrumentally for research-support functions.

This study reveals that early academic sentiment toward DeepSeek is overwhelmingly positive, especially in technical fields such as Computer Science and Engineering. The Word Heat Map confirms a strong focus on performance, model architecture, and practical use cases, with limited engagement in ethical or political critiques. Geographically, China and the United States lead in publication output, though global participation remains uneven. While early literature reflects a broadly positive perception of DeepSeek, a more comprehensive understanding of its scientific, ethical, and societal implications will require expanded interdisciplinary engagement and more regionally diverse research.
